# Prevalence and Risk Factors of Q Fever in Smallholder Dairy Farms in Kenya

**DOI:** 10.1002/vms3.71036

**Published:** 2026-06-29

**Authors:** Joseph Samuel Kimatu, Jackson Nyarongi Ombui, Timothy Muthui Wachira, Joseph Wasonga, Sylvia Cheptoo, Benson Rukwaro, Susan Migeni, Christine Mutisya, Getrude Nangekhe, Benedict Karani, Gideon Ndambuki, Erhan Yalcindag, Barend Mark de Clare Bronsvoort, Lina González Gordon, Elizabeth Anne Jessie Cook

**Affiliations:** ^1^ Department of Public Health, Pharmacology and Toxicology University of Nairobi, Kangemi Nairobi Kenya; ^2^ Center for Tropical Livestock Genetics and Health (CTLGH) International Livestock Research Institute (ILRI) Nairobi Kenya; ^3^ Department of Community Health Amref International University (AMIU) Nairobi Kenya; ^4^ Jomo Kenyatta University of Agriculture and Technology (JKUAT) Nairobi Kenya; ^5^ Department of Veterinary Pathology, Microbiology and Parasitology The University of Nairobi Nairobi Kenya; ^6^ Division of Epidemiology The Epidemiology, Economics and Risk Assessment (EERA) Group The Roslin Institute at the Royal (Dick) School of Veterinary Studies, University of Edinburgh Midlothian UK; ^7^ Center for Tropical Livestock Genetics and Health (CTLGH) The Roslin Institute at the Royal (Dick) School of Veterinary Studies, University of Edinburgh Midlothian UK

**Keywords:** abortion, cattle, *Coxiella burnetii*, prevalence, Q fever

## Abstract

**Background:**

Q fever is a neglected zoonotic disease of global concern. In Kenya, it ranks among the top priority zoonoses, yet data on its prevalence and associated risk factors in smallholder dairy farms are limited.

**Objectives:**

To estimate the seroprevalence and molecular prevalence of *Coxiella burnetii* and identify risk factors for exposure in dairy cattle on smallholder farms in Kenya.

**Methods:**

A cross‐sectional study was conducted on 448 smallholder dairy farms across Nandi County and nearby dairy‐producing areas. Serum (*n* = 1829), vaginal swabs (*n* = 1783) and preputial swabs (*n* = 52) were collected. Animal and herd‐level data on Q fever risk factors were recorded. An indirect IgG ELISA was used for antibody detection, whereas real‐time polymerase chain reaction (PCR) (quantitative PCR [qPCR]) was performed on the swab samples.

**Results:**

The animal‐level seroprevalence of *C. burnetii* was 8.7% (95% CI: 7.4–10.0), whereas herd‐level seroprevalence was 29.0% (95% CI: 24.8–33.2). Molecular prevalence detected by qPCR was 1.5% (95% CI: 0.9–2.0). Cows with a history of abortion had increased odds of seropositivity (OR = 1.73, 95% CI: 1.14–2.61). Cattle on farms where dogs were kept—whether fed placentas or not—had lower odds of seropositivity compared to farms without dogs.

**Conclusion:**

This study confirms circulation of Q fever in smallholder dairy herds in Kenya. Investigating environmental persistence, farm management, interactions between susceptible hosts and sociocultural aspects of dog ownership in smallholder settings is essential to unravel the local transmission dynamics of Q fever. These findings highlight the need for coxiellosis control strategies, including improved biosecurity, surveillance and evaluation of vaccination approaches that have been successfully applied in other endemic settings.

## Graphical abstract

Serological and molecular screening revealed widespread exposure to *Coxiella burnetii* in dairy cattle, linked to reproductive and herd management risk factors. Strengthened surveillance and herd‐level biosecurity are critical to reducing Q fever transmission.

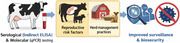

## 1 Introduction

The dairy industry in Kenya plays a fundamental role in enhancing nutrition and providing income for over 1.8 million smallholder farmers (Kiplagat et al. 2024). Despite significant progress, smallholder dairy farmers face numerous challenges that affect the productivity, profitability and sustainability of their activities (Duguma 2022). Limited access to quality fodder and feed resources (Hegde 2019), water scarcity (Muriuki 2003; Aguilar et al. 2022), and the high cost of veterinary and artificial insemination (AI) services contribute to inadequate nutrition, poor animal health and suboptimal breeding practices. High morbidity from endemic infections further exacerbates poor reproductive performance (Shija et al. 2022), resulting in substantial economic losses (Abebe et al. 2023), negatively impacting animal welfare and posing significant zoonotic risks (McElwain and Thumbi 2019).

Q fever is caused by the highly infectious gram‐negative, spore‐forming bacterium *Coxiella burnetii*, characterized by its resilience to environmental degradation and low infectious dose (Porter et al. 2011; Gürtler et al. 2013). Domestic ruminants, including cattle, sheep and goats, are the primary reservoirs of *C. burnetii* (Van den Brom et al. 2015; Theonest et al. 2021; Mwololo et al. 2022; Sadiki et al. 2023), though a wide variety of hosts, such as mammals, reptiles, rodents and birds, can be exposed through contaminated aerosols (Ullah et al. 2022).

In animals, transmission occurs primarily through contact with birth fluids and tissues from infected animals (Plummer et al. 2018; Zangue et al. 2022). It can also be sexually transmitted through infected semen from bulls, either directly or via AI (Johnson et al. 2019). Ticks may act as vectors and play an important role in the natural cycle of Q fever among livestock (Celina and Cerný 2022). Susceptible hosts can contract the disease through tick bites while grazing or by inhaling the bacterium from contaminated pastures (Maurin and Raoult 1999; Celina and Cerný 2022).

After exposure to *C. burnetii*, cattle mount an innate response, but intracellular survival permits persistent infection. *C. burnetii*‐specific IgM antibodies appear ∼1 and 2 weeks post exposure, followed by IgG antibodies from ∼3 to 4 weeks (Arricau‐Bouvery and Rodolakis 2005; Guatteo et al. 2011). During active infection, the organism concentrates in the placenta and mammary gland, causing heavy shedding in birth products, vaginal secretion, faeces, urine and milk for months to years after seroconversion, due to incomplete immune elimination and tolerance in reproductive tissues (Porter et al. 2011; Eldin et al. 2017). Q fever typically presents as a subclinical or asymptomatic infection in livestock (Ullah et al. 2022); however, infection in small ruminants is clinical, and in cattle, it can also lead to abortion, stillbirth, perinatal mortality and weak offspring as well as retained placenta, reproductive failure and metritis (Gisbert et al. 2024).

Humans can contract Q fever through inhaling aerosols from contaminated materials, consuming contaminated meat and unpasteurized dairy products, or through accidental exposure while handling contaminated fluids and tissues such as animal foetuses and afterbirth. Hence, abattoir workers, veterinarians and farmers are at higher risk through occupational contact (Cook et al. 2021; Ullah et al. 2022). In humans, infection is often asymptomatic but can also manifest as acute febrile illness or chronic conditions such as endocarditis, pneumonia and hepatitis (Maurin and Raoult 1999). Since 2015, Q fever has been listed as one of the top priority zoonoses in Kenya and is considered endemic, particularly in rural settings (Munyua et al. 2016). Besides direct costs from managing the outbreaks and indirect economic losses from reduced productivity and reproductive failures, dairy farmers face costs incurred from seeking healthcare and accessing treatment for symptomatic infections (Van Asseldonk et al. 2013; Raboisson et al. 2024).

Accurate diagnosis of Q fever requires specific laboratory testing (WOAH 2023). Antibody‐based tests only provide information on past exposure to infection and are limited by relatively poor specificity and inconsistent results (Rivière et al. 2025). Molecular techniques provide a more direct temporal link between the pathogen and clinical presentation (Shujat et al. 2024). Polymerase chain reaction (PCR) has become the WOAH diagnostic tool of choice, used as a standalone test for outbreak investigation, confirmation of suspected cases and as part of large‐scale epidemiological studies for disease monitoring or freedom of infection surveys (WOAH 2018). A relatively simple, fast and specific PCR and quantitative PCR (qPCR) targeting the *IS1111* element are considered the most sensitive methods for detection and quantification of *C. burnetii* (Klee et al. 2006). These methods allow the identification of *C. burnetii* from a variety of biological sample types, including vaginal swabs, faeces, semen, blood and plasma (Mares‐Guia et al. 2018; Orrego et al. 2020).

Although prior studies have documented the geographical patterns and seroprevalence of *C. burnetii* across susceptible hosts and livestock production systems in Kenya (Njeru et al. 2016; Grace and Watene 2021; Muturi et al. 2021; Kiptanui et al. 2022; Muema et al. 2022), the prevalence of *C. burnetii* in dairy cattle remains poorly documented. Most existing studies have relied on serological evidence alone, with the assays primarily detecting IgG antibodies. As IgG can persist for months to years after exposure (Mccaughey et al. 2010), seropositivity reflects past exposure rather than active infection, limiting inference on current infection status (Bento et al. 2023). Given the close human–animal interactions characteristic of smallholder dairy farms and the potential public health implications of ongoing bacterial shedding, there is a critical need for serological and molecular investigations. Additionally, *C. burnetii* dynamics are complex epidemiologically, with many factors involved not only regionally but also at the herd and animal level. Although previous studies have been conducted across Kenya, the shifting climatic conditions warrant the need for investigations of the changing dynamics and exposure of this zoonosis (Aldwekat et al. 2025). This study aimed to assess the seroprevalence, molecular prevalence, active infection (including bacterial shedding) and associated risk factors of *C. burnetii* in dairy cows within smallholder farms in Kenya. Farms in the study region were considered at high risk for Q fever due to the large livestock population and the sociocultural habits of farmers (Nandi County Integrated Development Plan 2023). The communities in this region value livestock, particularly cattle, as a prestige symbol and currency for dowry (Ndubi et al. 2025).

## 2 Materials and Methods

### 2.1 Study Area

**FIGURE 1:**
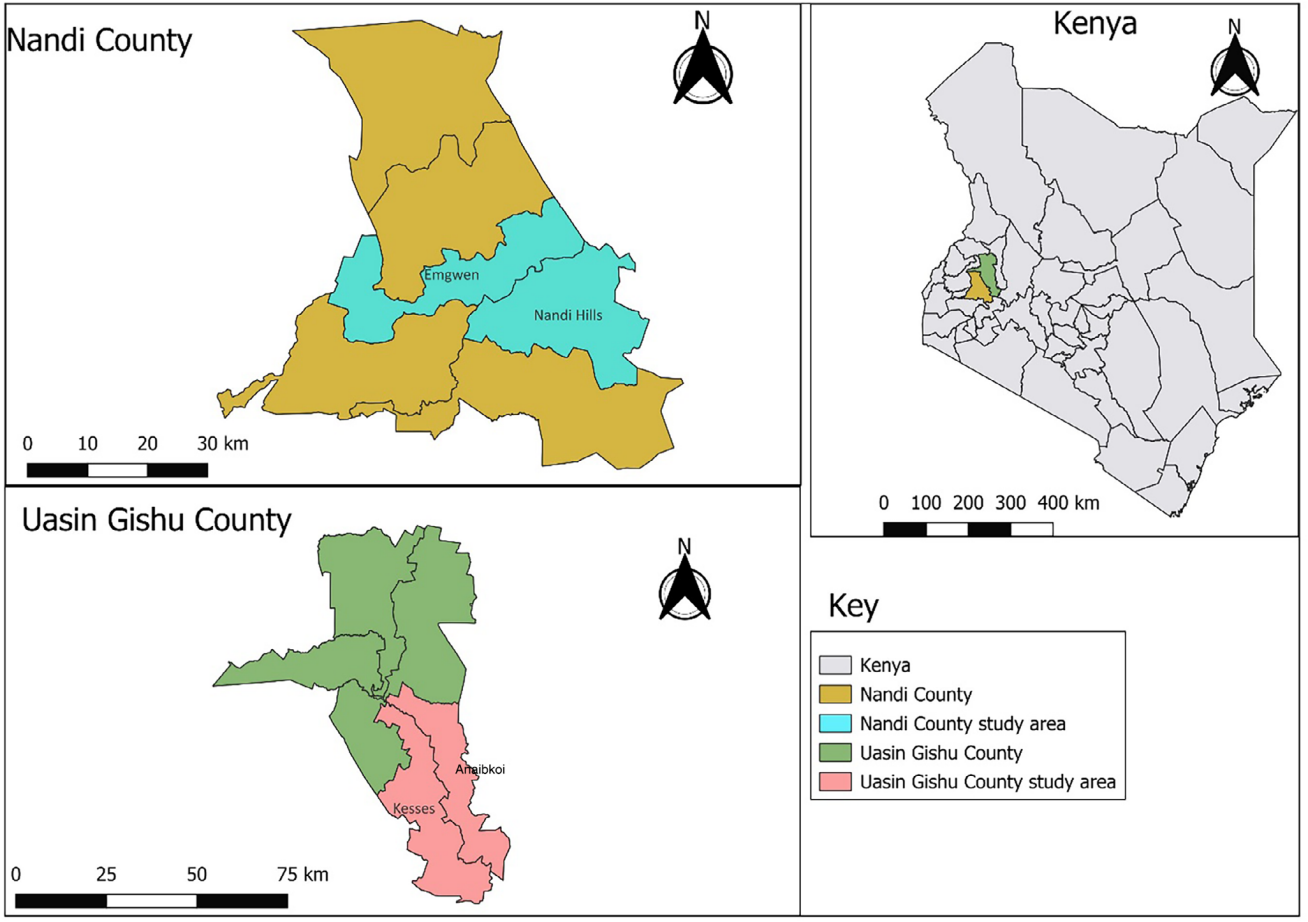
Map of Kenya *(top right)* showing location of Nandi and Uasin Gishu counties, and Nandi *(top left)* and Uasin Gishu *(bottom left)* county maps showing sub‐counties where sampling was conducted in Emgwen and Nandi Hills sub‐counties (Nandi County), Kesses and Anaibkoi sub‐counties (Uasin Gishu County). Map created from GIS version 3.36.0. Shapefiles downloaded from Humanitarian Data Exchange (https://data.humdata.org/dataset/cod‐ab‐ken).

This study was carried out on dairy farms belonging to the Lessos Dairy Farmer Cooperative primarily operating in Nandi County (Emgwen and Nandi Hills sub‐counties) but also at the border in Uasin Gishu County (Kesses and Anaibkoi sub‐counties) (Nandi County Integrated Development Plan 2023). These counties are located in the Rift Region of Kenya, situated between latitudes 0.56° N and 0.11° S and longitudes 34.05° E–35.03° E (Figure 1).

The study area is at an altitude ranging from 1300 to 2500 m above sea level, with average daily temperatures between 18°C and 22°C and an annual rainfall of 1200–2000 mm. Historically, there has been a bimodal rainfall pattern, with rainy seasons from March to June and September to December, although recently these patterns are not consistent. The intensity and distribution of rainfall in these counties have a direct impact on economic activities, particularly dairy farming as the conditions are favourable for fodder production.

### 2.2 Study Design

A cross‐sectional study was conducted on cattle on smallholder dairy farms between September and December 2023. The sampling frame was the baseline cohort for a large‐scale longitudinal study of abortions led by the Centre for Tropical Livestock Genetics and Health (CTLGH) (https://www.ctlgh.org/) among farms registered with the Africa Dairy Genetic Gains (ADGG) programme (https://hdl.handle.net/10568/128305). A total of 448 farms with a median of 3 adult animals were included. Data on potential risk factors for Q fever were collected using a structured questionnaire that was developed, piloted and administered to animal owners or their representatives aged ≥18 years old. Prior to participation, respondents were informed of the study objectives and provided written informed consent. Interviews were conducted in Kiswahili or local vernacular to ensure clarity and accuracy. The questionnaire captured information on the general background of the interviewee, as well as farm location, herd management practices, presence of other animals on the farm or in the neighbourhood, handling and disposal of abortion or birth materials, and history of abortions. Animal‐level information was captured and samples (serum and genital swabs) collected (Table S1). Responses were recorded using the Open Data Kit (ODK) application and uploaded to a secure server hosted by International Livestock Research Institute (ILRI), using the ONA platform (https://opendatakit.org).

### 2.3 Sample Collection and Processing

Samples were collected from dairy cattle, specifically bulls and females that had ever been served as assessed by a trained veterinarian. Animals were restrained, and 10 mL whole blood was collected by jugular venipuncture with a sterile needle into a serum vacutainer tube coated with a clot activator (Becton, Dickinson and Company, NJ, USA). Vaginal swabs were collected after cleaning the perineal area, using clean gloved hands and sterile swabs. The swab was rotated at least 10 times against the vaginal mucosa. Preputial swabs were obtained by gently inserting and rotating the swab in the preputial cavity to gather cellular material and secretions. Swabs were thoroughly mixed with phosphate‐buffered saline (PBS) for storage. Samples were transported the same day in ice‐filled cool boxes to Nandi County Veterinary laboratory. Blood tubes were centrifuged at 3000 rpm for 15 min, and serum was aliquoted into 2 mL cryovials for storage at −20°C.

### 2.4 Serological Analysis

Serum samples were screened for anti‐*C. burnetii* IgG antibodies, indicative of exposure that many have occurred months to years prior to sampling, using the ID Screen Q Fever Indirect Multispecies ELISA kit (Innovative Diagnostics, Montpellier, France). The test was performed according to the manufacturer's instructions. Samples were classified as negative, doubtful, positive or strong positive on the basis of the sample‐to‐positive ratio (S/P%) thresholds provided in the kit manual. The optical density (OD) was measured at 450 nm using an ELISA microplate reader, and test validity was confirmed using the provided positive and negative controls. The S/P% was calculated using the formula: S/P% = (OD_s_/OD_pcl_ × 100), where S means serum sample, P means positive sample, OD_s_ is the OD of the sample, and OD_pc_ is the OD of the positive control. Samples were considered positive if the S/P ratio was greater than 80% (strong positive).

### 2.5 Molecular Analysis of Vaginal and Preputial Swabs

DNA from vaginal and preputial swabs was extracted using the TANBead Nucleic Acid Extractor (Maelstrom 9600) with the TANBead Nucleic Acid Extraction Kit (W6T2A46) (https://www.tanbead.com/en) according to the manufacturer's instructions. A qPCR assay targeting the *Coxiella*‐specific, multi‐copy transposase gene in the insertion element *IS1111* sequence was performed using *IS1111* forward primer (5′‐CATCACATTGCCGCGTTTAC‐3′), *IS1111* reverse primer (5′‐GGTTGGTCCCTCGACAATCAT‐3′) and a dual labelled fluorescent probe (5′‐FAM‐AATCCCCAACAACACCTCCTTATTCCCAC‐BHQ‐1‐3′) (Roest et al. 2011; de Bruin et al. 2012).

The reaction mix included 10 µL of Luna Universal Probe qPCR Master Mix (1X with UDG; 404; New England BioLabs, MA, USA), 5 µL of DNA template, 0.8 µL of each *IS1111* forward primer and reverse primer (final concentration 0.4 µM for both), 0.4 µL of FAM‐labelled probe (final concentration 0.2 µM) and 3.0 µL of nuclease‐free water, making a total reaction volume of 20 µL per well, according to the manufacturer's instructions. Each qPCR plate included a synthetic DNA sequence of *C. burnetii* DNA cloned into a plasmid as a positive control and nuclease‐free water as a negative control to monitor for cross‐contamination. The plates were sealed, centrifuged and analysed using the QuantStudio 5 system (Applied Biosystems). The qPCR thermocycling conditions consisted of an initial denaturation step at 95°C for 60 s, followed by amplification cycles, each comprising denaturation at 95°C for 15 s and extension at 60°C for 30 s, during which fluorescence was measured, according to the manufacturer's instructions. A run was considered valid only if the positive controls amplified and the negative controls showed no amplification. Samples with a cycle threshold (Ct) value of ≤40 were considered positive (Orrego et al. 2020).

### 2.6 Data Management and Analysis

All data analyses were conducted in RStudio (version 4.4.2) using R statistical software (https://www.R‐project.org/). Crude animal and farm‐level seroprevalence and survey‐weighted proportions adjusted for clustering at the herd level were calculated using *svydesign* and *svymean* functions from the *survey* package (Lumley 2024).

**FIGURE 2:**
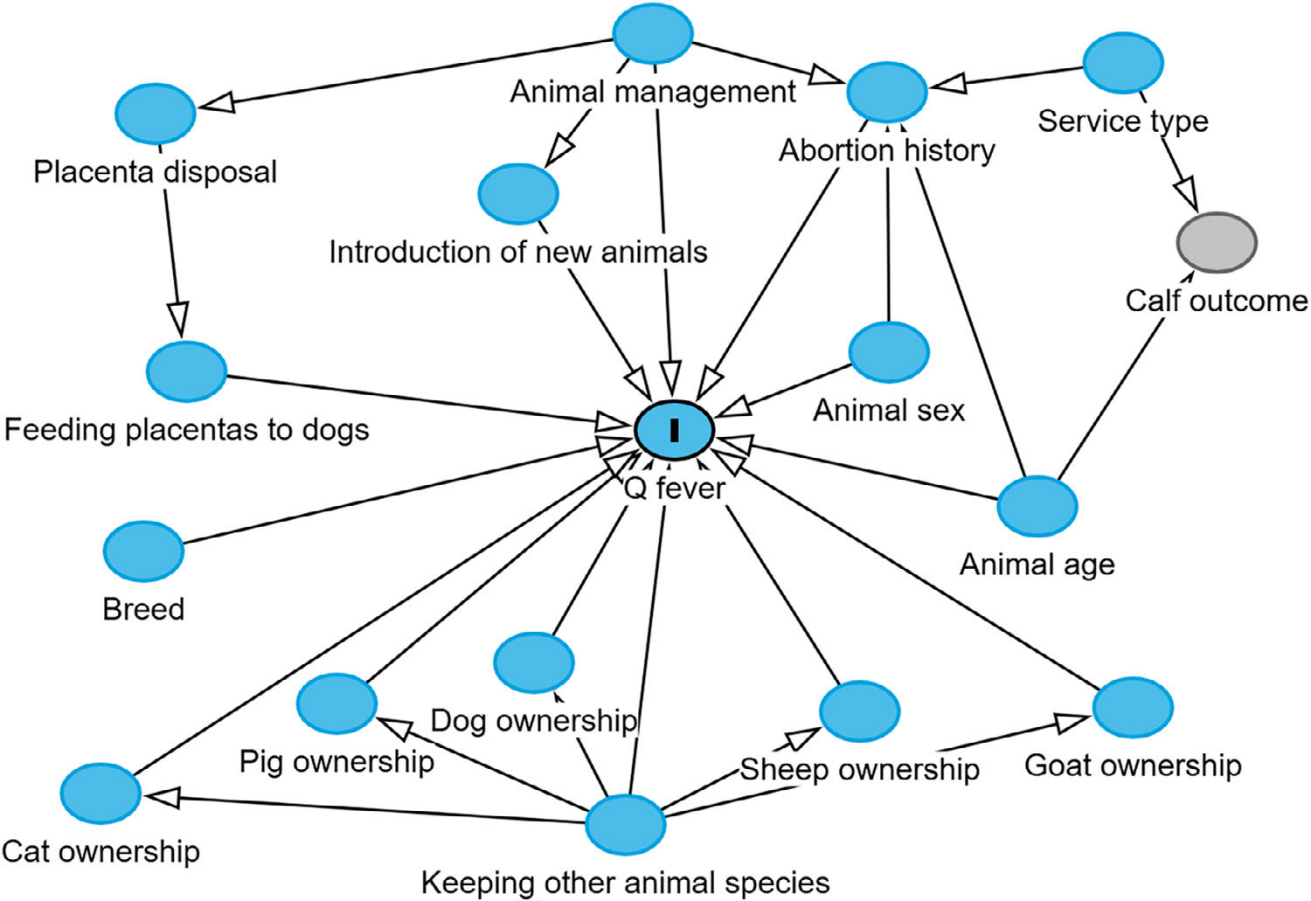
Directed acyclic graph (DAG) of hypothesized causal pathways linking farm and animal‐level factors to Q fever status and downstream calf outcome. Blue nodes are measured exposures; the grey node is the calf outcome. Arrows indicate assumed causal direction. The central Q fever node represents infection/serostatus assessed in this study by PCR (current infection) and ELISA (past exposure); edges into Q fever denote putative risk factors (management practices, abortion history, animal demographics, biosecurity and contact with other species), and the edge from Q fever to calf outcome represents potential effects of infection on pregnancy outcomes. The DAG was created from DaGitty v3.1 (https://www.dagitty.net/).

To assess risk factors for *C. burnetii* seropositivity in smallholder dairy cattle, both univariable and multivariable mixed‐effects logistic regression models were fitted using the *glmer* function from the *lme4* package (Bates et al. 2015), excluding records with missing data. Explanatory variables included individual animal‐level factors (age, sex, breed [Ayrshire cross, Channel Island cross and Holstein Friesian cross], service type, history of abortion, calf outcome and introduction of new animals) and herd‐level factors (placenta disposal method, management system [mixture of grazing pasture and cut and carry, pasture only, zero grazing], presence of other livestock species, feeding placenta to dogs and ownership of cats, goats, sheep or pigs). Variables were selected on the basis of biological plausibility and prior evidence (Figure 2).

Multivariable logistic regressions model building used backward stepwise selection from variables with *p* ≤ 0.2 in the univariable analysis. Covariates were retained if their inclusion reduced the Akaike information criterion (AIC) and improved model fit (*p* ≤ 0.05). Farm was included as a random effect, and animal age was retained as a potential confounder.

## 3 Results

### 3.1 Descriptive Statistics

The study population consisted of 1837 dairy cattle, including 1785 females (97.2%) and 52 (2.8%) males. From these, 1829 serum samples (1777 from female animals and 52 from males) and 1835 swabs (1783 vaginal and 52 preputial) were available for screening. Descriptive statistics on animal and farm‐level variables are presented in Table 1. On the basis of farmer‐reported breed composition, Holstein Friesian crosses formed the highest per cent of the study population (61.0%). Over half (53.3%) of the farms reported animals being born on the farm, and most of the cattle (45.1%) were reported to be fed with a mix of pasture grazing and cut and carry. Additionally, 55.6% of the farms kept other livestock species. Dairy farming was the primary source of income for 37.3% of farmers (167/448).

**TABLE 1:** Descriptive statistics of the study population relative to individual and farm‐level variables.

Variables	Category	Number (*n*)	Percentage
**Farm level (*n* = 448)**			
Farmer education	None	7	1.6
	Primary	86	19.2
	Secondary	260	58.0
	Tertiary	95	21.2
Farmer gender	Female	203	45.3
	Male	245	54.7
Role of the herd to the farmer	Primary income	167	37.3
	Secondary income	281	62.7
Keeping other animal species	No	199	44.4
	Yes	249	55.6
Cat ownership	No	31	6.9
	Yes	417	93.1
Sheep ownership	No	56	12.5
	Yes	392	87.5
Goat ownership	No	359	80.1
	Yes	89	19.9
Pig ownership	No	433	96.7
	Yes	15	3.3
Placenta disposal	Bury	108	24.1
	Not bury	340	75.9
Feeding placentas to dogs	Does not own dogs	141	31.5
	Owns dogs and feeds placentas	240	53.6
	Owns dogs and does not feed placentas	67	14.9
Introduction of new animals	No	239	53.3
	Yes	209	46.7
History of abortion	No	389	86.8
	Yes	59	13.2
**Animal level**			
Animal management (*n* = 1829)	Mixture of grazing pasture and cut and carry	824	45.1
	Pasture only	780	42.6
	Zero‐graze only	225	12.3
Animal sex (*n* = 1829)	Female	1777	97.2
	Male entire	52	2.8
Animal breed (*n* = 1829)	Ayrshire cross	631	34.5
	Channel Island cross	83	4.5
	Holstein Friesian cross	1115	61.0
Service type (*n* = 1752)	Artificial insemination	693	39.6
	Natural	1059	60.4
Cow ever calved (*n* = 1752)	No	266	15.2
	Yes	1486	84.8
Calf outcome (last calving) (*n* = 1486)	Alive	1422	95.7
	Dead	64	4.3

With respect to reproduction and abortion‐related factors, 60.4% of the cattle were bred naturally using bulls, and 13.2% of farmers reported experiencing an abortion in the herd within the previous 12 months.

Farmers reported using various methods of placenta disposal, with 75.9% of them either feeding placentas to dogs or disposing of them in rubbish heaps. Regarding dog ownership and feeding practices, 53.6% of the farmers owned dogs and fed them placentas.

### 3.2 Seroprevalence of *C. burnetii*

A total of 159 serum samples tested positive for *C. burnetii* antibodies (*n* = 1829), resulting in an adjusted seroprevalence at the herd level of 8.7% (95% CI: 7.4–10.0). Of the 159 positive samples, 154 were from females originating from 127 farms and with a mean age of 40 months, whereas the 5 male positives were aged 12–36 months and came from 5 different farms. Of the 448 farms sampled, 130 had at least one seropositive animal, corresponding to a herd‐level seroprevalence of 29.0% (95% CI: 24.8–33.2).

### 3.3 Univariable Analysis of Factors Associated With *C. burnetii* Antibody Seropositivity

Univariable analyses were restricted to female animals (*n* = 1777) because reproductive variables are only applicable to females and the number of males was small (*n* = 52). In the univariable analyses (Table 2), cows with a history of abortion had higher odds of being seropositive than cows without abortion reports (OR = 1.67, 95% CI: 1.09–2.56). Considering dog ownership and the practice of feeding placentas to dogs, dairy cattle kept in farms that owned dogs and fed placentas to them had lower odds of seropositivity compared to cattle kept in households without dogs (OR = 0.53, 95% CI: 0.37–0.76). Breeding methods, recent calving and animal‐level factors, such as age and breed, showed no significant statistical association with seropositivity (*p* > 0.05; Table 2 and Table S2).

**TABLE 2:** Univariable analysis of risk factors for Q fever seropositivity in dairy cattle.

Variables	Category	Number of observations	%Q fever positive (*n*)	Odds ratio (95% CI)	*p* value
**Animal contacts and management practices**					
Keeping other animal species	No	705	9.1 (64)	Ref.	
	Yes	1072	8.4 (90)	0.92 (0.64–1.32)	0.650
Feeding placentas to dogs	Does not own dogs	463	12.5 (58)	Ref.	
	Owns dogs and fed placenta	1073	7.1 (76)	0.53 (0.37–0.76)	**0.001**
	Owns dogs and does not feed placentas	241	8.3 (20)	0.63 (0.37–1.08)	0.092
Cat ownership	No	87	9.2 (8)	Ref.	
	Yes	1690	8.6 (146)	0.94 (0.43–2.06)	0.877
Sheep ownership	No	201	9.0 (18)	Ref.	
	Yes	1576	8.6 (136)	0.94 (0.54–1.63)	0.829
Goat ownership	No	1429	8.5 (122)	Ref.	
	Yes	348	9.2 (32)	1.09 (0.71–1.69)	0.684
Pig ownership	No	1698	8.6 (146)	Ref.	
	Yes	79	10.1 (8)	1.15 (0.51–2.63)	0.735
**Reproductive history**					
History of abortion—animal level	No	1496	8.0 (119)	Ref.	
	Yes	281	12.5 (35)	1.67 (1.09–2.56)	**0.019**
Service type (*n* = 1752)	Artificial insemination	693	8.9 (62)	Ref.	
	Natural	1059	8.5 (90)	0.95 (0.66–1.35)	0.757
Calf outcome last calving (*n* = 1486)	Alive	1422	9.4 (133)	Ref.	
	Dead	64	6.3 (4)	0.65 (0.23–1.83)	0.410

Bold values indicate significantly significant associations (*p* < 0.05).

### 3.4 Multivariable Analyses of Risk Factors Associated With Seropositivity in Dairy Cattle

The final multivariable logistic regression model included 1777 female animals from 448 farms, after excluding 52 males. Of the variables included from the univariable models, only the history of abortion, owning dogs and feeding them placentas, and owning dogs and not feeding them placentas were retained as statistically significant factors in the final model (Table 3). Cattle kept in farms where dogs were fed placentas had lower odds of testing seropositive for *C. burnetii* compared to cattle kept in farms without dogs being present (OR = 0.53, 95% CI: 0.37–0.77). Similarly, cattle kept in farms where dogs were not fed placentas were also less likely to be seropositive (OR = 0.55, 95% CI: 0.32–0.95). Cattle that had experienced an abortion in the last 12 months were 1.73 (OR = 1.73, 95% CI: 1.14–2.61) times more likely to be seropositive than those with no abortion record. Animal age was maintained in the multivariable model to control for confounding, although not statistically significant *(p* > 0.05*)*.

**TABLE 3:** Multivariable mixed‐effect logistic regression model of risk factors for Q fever seropositivity in dairy cattle.

Variables	Category	Odds ratio (95% CI)
Animal age (months)	12–36	Ref.
	37–53	0.95 (0.59–1.54)
	54–72	1.28 (0.82–1.99)
	73–180	0.83 (0.50–1.38)
History of abortion—animal level	No	Ref.
	Yes	1.73 (1.14–2.61)
Dogs fed placenta	Does not own dogs	Ref.
	Owns dogs and fed them placentas	0.53 (0.37–0.77)
	Owns dogs and did not feed them placentas	0.55 (0.32–0.95)

### 3.5 Vaginal and Preputial Shedding of *C. burnetii*

A total of 1835 DNA samples extracted from 1783 vaginal swabs and 52 preputial swabs were tested for the presence of *C. burnetii* using qPCR. Of the tested samples, 27/1835 tested positive (Table S3). The animal‐level prevalence, adjusted for clustering, was 1.5% (95% CI: 0.9–2.0) based on the qPCR results. Among the 27 positive samples, one was from a male animal, whereas 26 were from female animals. A total of 25 out of 448 farms had at least one positive animal, resulting in a herd‐level prevalence of 5.6% (95% CI: 3.5–7.7). Among 159 ELISA‐seropositive cattle, *C. burnetii* was detected in only 2 samples.

Comparison of qPCR and ELISA results (Table 4) showed that 1644 (89.9%) animals were negative on both tests, whereas 2 (0.1%) were positive on both. Twenty‐five animals (1.4%) were qPCR positive and ELISA negative. A total of 157 (8.6%) animals were ELISA positive and qPCR negative. Agreement between ELISA and qPCR was assessed using Cohen's kappa statistic, and there was no agreement beyond chance (*k* = 0.0).

**TABLE 4:** Comparison of polymerase chain reaction (PCR) and ELISA test results.

	PCR		
ELISA	Negative (%)	Positive (%)	Total (%)
Negative (%)	1644 (89.9)	25 (1.4)	1669 (91.3)
Positive (%)	157 (8.6)	2 (0.1)	159 (8.7)
**Total**	1801 (98.5)	27 (1.5)	**1828**

## 4 Discussion

The current study investigated seroprevalence and molecular detection of *C. burnetii* in dairy cattle from smallholder dairy farms. Exposure to Q fever was found to be widespread, and active shedding of the pathogen was detected, with low agreement between serology and qPCR results. History of abortion was a significant factor, whereas the presence of dogs on farms showed an unexpected inverse association. These findings improve the understanding of Q fever epidemiology in smallholder dairy settings and can guide control efforts.

*C. burnetii* has a worldwide distribution and is endemic in many African countries, where it is frequently associated with human outbreaks and is classified as a neglected zoonosis (Tan et al. 2024). As key disease reservoirs, domestic ruminants pose a considerable risk to human populations, especially in smallholder farming communities, who may have an increased risk of exposure through different routes (Pouquet et al. 2020; Konputtar et al. 2024; Tan et al. 2024). This study contributes to the body of research on Q fever in Kenya by investigating both serological and molecular prevalence of *C. burnetii* in dairy cattle. The seropositivity of 8.7% and qPCR prevalence of 1.5% in genital swabs underscore the significance of the disease in this setting, posing both a public health threat and potentially hindering livestock productivity in the region (Kiptanui et al. 2022).

Reported seroprevalence of Q fever in Africa is highly heterogeneous, varying by livestock system and species (Vanderburg et al. 2014; Salifu et al. 2019). In dairy cattle, seroprevalence ranges from approximately 4% to 6.2% in studies conducted in Eastern Africa, including in smallholder farms in Tanzania (Bwatota, Shirima et al. 2022) and Ethiopia (Robi and Gelalcha 2020). A study by Kiptanui et al. (2022) on domestic ruminants in Nandi reported a cattle seroprevalence of 8.14% which aligns with our results with higher levels of exposure in cattle compared to small ruminants (Kiptanui et al. 2022). Multiple studies have highlighted the widespread distribution of this pathogen in livestock and humans across agroecological zones, herding systems and at different wildlife‐livestock interfaces in Kenya. The highest seroprevalence has been documented in goats (up to ∼75%), whereas it is moderate in sheep (up to ∼57%), cattle (up to ∼52%) and camels (up to ∼46%) (Njeru et al. 2016; Larson et al. 2019; Muema et al. 2022; Wambua et al. 2025), which might indicate the potential for cross‐species transmission during co‐farming, a common practice among smallholders.

Growing evidence of the role of *C. burnetii* infection in reproductive outcomes in cattle underscores the need for rapid identification and effective management of infected animals (Gisbert et al. 2024). Studies from East Africa and beyond report significant associations between Q fever seropositivity and a history of abortion in dairy cattle consistent with our findings (Bwatota, Cook et al. 2022; Hussain et al. 2022; Watene et al. 2022; Elkhaiat et al. 2024). Aside from abortion, *C. burnetii* has been linked to several reproductive issues, resulting in substantial but preventable economic losses for dairy producers (Gisbert et al. 2024).

In many rural settings, Q fever remains poorly recognized by farmers, who often have limited knowledge of the disease, low‐risk perception and minimal engagement in disease surveillance, contrasting sharply with the priorities of Kenyan veterinary and public health authorities (Munyua et al. 2016; Zangue et al. 2022). Disease control is further challenged by the limited sensitivity of available diagnostic tests, the difficulty of treating cases and the circulation of multiple abortifacient pathogens in dairy farms (Plummer et al. 2018; Rivière et al. 2025). In several countries, vaccination against *C. burnetii* has been implemented successfully using licensed vaccines such as the inactivated Phase 1 vaccines which have demonstrated effectiveness in reducing infection (Gisbert, Hurtado, et al. 2024). Although vaccination offers a promising yet not widely available strategy to mitigate both direct and indirect disease impacts (Plummer et al. 2018; Williams‐Macdonald et al. 2023), large‐scale vaccine deployment in rural contexts remains constrained by lack of licensure for use and unavailability, a challenge observed in Nandi as well. Future work should quantify the burden of Q fever on dairy systems by estimating milk production losses, effects on calving intervals and other key reproductive indicators (Gisbert et al. 2024; Raboisson et al. 2024), while also assessing the effect of mixed interventions combining enhanced farmer awareness to improve disease surveillance and vaccination.

Widespread biosecurity failures in smallholder farms increase the risk of disease transmission and the persistence of highly environmentally resistant pathogens, such as *C. burnetii*. Dog keeping, as a fluid and diverse practice across rural settings, can contribute to disease spread, particularly in environments where roaming dogs are common (Murungi et al. 2025; Tayebwa et al. 2025). Equally important are practices, such as feeding placentas to dogs, which are discouraged from a biosecurity perspective but remain frequent among smallholders. The lower seroprevalence of *C. burnetii* among farms that keep dogs, regardless of whether placentas are fed, is puzzling and counterintuitive compared to existing literature, which often identifies dogs as potential amplifiers of infection (Deressa et al. 2020; Bauer et al. 2021). These findings may reflect environmental or management‐related factors that reduce exposure to infectious birth material or indicate shifts in the relevance of domestic hosts in Q fever endemic environments as previously hypothesized in a study examining dog–cattle seropositivity dynamics in Ecuador (Guerrero‐Freire et al. 2024). The unmeasured confounders related to farm size or husbandry practices could also influence both dog presence and infection risk. In addition, respondents involved in this study could have interpreted the question about dogs in different ways, hence indicating less risk in farms that kept dogs. Holistic animal and public health interventions should, therefore, account for cultural and behavioural practices that could drive disease spread, including education on the risks associated with improper management and disposal of afterbirth tissues, as these practices can increase *C. burnetii* transmission.

The detection of actively infected animals via qPCR on vaginal and preputial swabs raises significant concerns as these animals pose a risk to other livestock, farm workers and household members. Infected cattle shed *C. burnetii* through milk, faeces and birth products, contaminating the environment and enabling aerosol transmission to humans and other animals, even at minimal doses (Alemneh 2018). Farm workers are particularly at risk during calving and milking (Guidi et al. 2017), whereas household members may also be exposed through consumption of raw milk (Pexara et al. 2018). Screening cattle for Q fever may not be economically feasible for dairy farmers in this region, and the uptake of interventions to reduce transmission of infectious diseases remains low. Even basic hygiene and biosecurity practices around calving and abortion events are often neglected, despite their potential to prevent not only Q fever but also other prevalent infections, zoonotic or otherwise. Farmers should be advised on routes of transmission and preventive measures.

The molecular prevalence detected here (1.5%) is lower than reports from other regions. A meta‐analysis of studies in Africa estimated a pooled *C. burnetii* prevalence of 9% in cattle on the basis of antigen and molecular data, with marked regional variations: Algeria (10%), Kenya (2%), Zambia (8%) and Tanzania (23%), alongside higher estimates in goats (23%) and sheep (16%) (Bwatota, Cook et al. 2022). Similarly, a study conducted in South Africa reported a PCR prevalence of 15.67% in cattle (Sadiki et al. 2023), whereas Mangena et al. (2021) also found a PCR prevalence of 12.7% in slaughter livestock. Although these studies did not explicitly report concurrent outbreaks or provide detailed seasonal stratification, higher prevalence in slaughter animals and semi‐arid regions suggests possible influences of animal movement, market dynamics or variations in management practices. Grazing practices may also affect *C. burnetii* detection as shedding dynamics increase under environmental stress and are influenced by housing and reproductive cycles. Continuous grazing may as well increase exposure to tick bites, thereby influencing the risk of tick‐borne infection and its detection (Ringa‐Ošleja et al. 2023).

The challenge of detecting and interpreting the findings of diagnostic tests for coxiellosis in farms is exemplified by the 8.6% of the animals that were ELISA‐positive but qPCR‐negative, suggesting past exposure to *C. burnetii* with no active shedding via vaginal mucus or preputial discharge. Subclinical infection with *C. burnetii* is far more common than clinical disease, making it difficult to determine individual and farm status for decision‐making and therapeutic planning (Plummer et al. 2018). Although some animals recover from acute infections, others may excrete *C. burnetii* in faeces, milk and vaginal secretions for several days to months postpartum (Guatteo et al. 2006). Two animals were positive by both qPCR and ELISA, indicating active bacterial shedding coupled with seroconversion. Conversely, 25 animals were qPCR‐positive but ELISA‐negative, likely reflecting recent infections where antibodies had not yet developed (Anastácio et al. 2016; Barkallah et al. 2018; Menadi et al. 2022). These findings underscore an important limitation of serological testing that detects only IgG antibodies. Seronegative animals may actively shed *C. burnetii* particularly during short windows with vaginal shedding being transient and easily missed (Guatteo et al. 2012). Of particular, importance is the detection of *C. burnetii* in one preputial swab, demonstrating the potential for transmission to cows through AI or natural breeding, particularly when bulls are shared across multiple farms. *C. burnetii* has been detected in semen and male reproductive tissues and other species (Yatsentyuk et al. 2019), and venereal transmission through natural mating has been proposed as a plausible route of herd‐level spread (Astobiza et al. 2011). In smallholder systems where natural service is widely practised and bulls often service multiple cows, even infrequent shedding by males may facilitate transmission (Angelakis and Raoult 2010). Although the limited intact males in this study precluded inclusion in multivariable analyses, the observed molecular positivity underscores the need to consider breeding males in Q fever surveillance and control strategies.

Local protocols should, therefore, prioritize health screening alongside genetic evaluation and implement quarantining of newly purchased bulls, as recommended by international standards, though confirming *C. burnetii* remains challenging (Givens 2018; Plummer et al. 2018). The absence of a significant association between ELISA and qPCR results further indicates that seropositivity did not consistently correspond with active detection of *C. burnetii* DNA, likely reflecting differences in the diagnostic targets of the two assays and the temporal dynamics of infection.

## 5 Conclusion

Q fever is actively circulating among smallholder dairy systems in Kenya and is associated with reproductive losses in cattle. Although the role of dogs in reducing or facilitating transmission remains unclear, these findings underscore the need for enhanced farmer education, routine diagnostics and One Health surveillance approaches to prevent human infections and economic losses. Prompt removal and disposal of aborted foetuses and placental material, proper cleaning and disinfection of calving areas, and minimizing dust and manure build‐up represent low or no‐cost interventions to curtail disease spread, whereas use of personal protective equipment (PPE) is crucial to minimize the risk of occupational exposure. In addition, separating periparturient animals from pregnant and young stock can further reduce transmission risk within farms. Targeted control strategies focusing on improving farm hygiene, managing breeding animals carefully and raising farmer awareness are essential to reduce the burden of Q fever in smallholder cattle systems.

## Author Contributions

Conceptualization: **Joseph Samuel Kimatu**, **Jackson Nyarongi Ombui**, **Timothy Muthui Wachira**, **Barend Mark de Clare Bronsvoort** and **Elizabeth Anne Jessie Cook**. Methodology: Joseph Samuel Kimatu, Barend Mark de Clare Bronsvoort and Elizabeth Anne Jessie Cook. Software: Joseph Samuel Kimatu, Barend Mark de Clare Bronsvoort and Elizabeth Anne Jessie Cook. Data curation: Joseph Samuel Kimatu, **Joseph Wasonga**, **Sylvia Cheptoo**, **Benson Rukwaro**, Barend Mark de Clare Bronsvoort, **Lina González Gordon** and Elizabeth Anne Jessie Cook. Investigation: Joseph Samuel Kimatu, Joseph Wasonga, Sylvia Cheptoo, Benson Rukwaro, **Susan Migeni**, **Christine Mutisya**, Getrude Nangekhe, **Benedict Karani**, Gideon Ndambuki, **Erhan Yalcindag**, Barend Mark de Clare Bronsvoort and Elizabeth Anne Jessie Cook. Validation: Joseph Samuel Kimatu, Barend Mark de Clare Bronsvoort, Lina González Gordon and Elizabeth Anne Jessie Cook. Formal analysis: Joseph Samuel Kimatu, Lina González Gordon, Barend Mark de Clare Bronsvoort, Lina González Gordon and Elizabeth Anne Jessie Cook. Supervision: Jackson Nyarongi Ombui, Timothy Muthui Wachira, Barend Mark de Clare Bronsvoort and Elizabeth Anne Jessie Cook. Funding acquisition: Barend Mark de Clare Bronsvoort and Elizabeth Anne Jessie Cook. Visualization: Joseph Samuel Kimatu, Gideon Ndambuki, Erhan Yalcindag, Barend Mark de Clare Bronsvoort, Lina González Gordon and Elizabeth Anne Jessie Cook. Project administration: Barend Mark de Clare Bronsvoort and Elizabeth Anne Jessie Cook. Resources: Barend Mark de Clare Bronsvoort and Elizabeth Anne Jessie Cook. Writing – original draft: Joseph Samuel Kimatu, Barend Mark de Clare Bronsvoort, Lina González Gordon and Elizabeth Anne Jessie Cook. Reviewing and editing: Joseph Samuel Kimatu, Jackson Nyarongi Ombui, Timothy Muthui Wachira, Joseph Wasonga, Sylvia Cheptoo, Benson Rukwaro, Susan Migeni, Christine Mutisya, Gideon Ndambuki, Benedict Karani, Gideon Ndambuki, Erhan Yalcindag, Barend Mark de Clare Bronsvoort, Lina González Gordon and Elizabeth Anne Jessie Cook.

## Funding

Authors acknowledge that this research was funded in part by the Bill & Melinda Gates Foundation and with UK aid from the UK Foreign, Commonwealth and Development Office (Grant Agreement INV‐040641) under the auspices of the Centre for Tropical Livestock Genetics and Health (CTLGH), established jointly by the University of Edinburgh, SRUC (Scotland's Rural College) and the International Livestock Research Institute. The findings and conclusions contained within are those of the authors and do not necessarily reflect the positions or policies of the Bill & Melinda Gates Foundation nor the UK Government. Research at the Roslin Institute was funded through support from the UK Biotechnology and Biological Sciences Research Council (BBS/E/RL/230002C; BBS/E/RL/230002D—B.B., E.Y.).

## Ethics Statement

Ethical approval for this study was sought from the University of Nairobi Faculty of Veterinary Medicine Biosafety at the Animal Use and Ethics Committee (approval number: FVM BAUEC/2023/525), Institutional Ethics Review Committee (Approval number: ILRI‐IREC2023‐44) and Institutional Animal Care and Use Committee (approval number: ILRI‐IACUC2023‐10) at the International Livestock Research Institute (ILRI). The University of Edinburgh Human Ethical Review Committee (approval number: HERC_2023_117) and The Animal Welfare Ethical Review Body (approval number: OS06‐23) also approved this study. A research licence was obtained from the National Commission for Science, Technology and Innovation (licence number: NACOSTI/P/24/39275).

## Conflicts of Interest

The authors declare no conflicts of interest.

## Supporting information




**Table S1**: Structured questionnaire for household, herd and animal‐level data collection in smallholder dairy farms.
**Table S2**: Additional Univariable analysis of risk factors for Q fever seropositivity (*n* = 1777).
**Table S3**: qPCR positive samples with their respective CT values.

## Data Availability

The data supporting the findings of this study will be made available by the corresponding author upon reasonable request and following publication.
