# Supplementary material for: Prevalence and Risk Factors of Q Fever in Smallholder Dairy Farms in Kenya
Source: Vet Med Sci. 2026 Jun 29;12(4):e71036. doi: 10.1002/vms3.71036 (PMC13312989; doi:10.1002/vms3.71036)
Supplement: Supplementary file 1 — Table S1: Structured questionnaire for household, herd and animal‐level data collection in smallholder dairy farms. Table S2: Additional Univariable analysis of risk factors for Q fever seropositivity (n = 1777). Table S3: qPCR positive samples with their respective CT values. [file VMS3-12-e71036-s001.docx]

**Prevalence and risk factors of Q fever in smallholder dairy farms in Kenya**

**Supplementary Table S1:** Structured questionnaire for household, herd, and animal-level data collection in smallholder dairy farms

| **Sections of the Questionnaire** | |
| --- | --- |
| Survey metadata | Interview date, start and end time.  Data collector and farmer identification. |
| Consent | Farmer consent to participate and to allow sampling. |
| Interviewee profile | Participant ID, role in cattle management, gender, education level, years of experience.  Importance of cattle to the household. |
| Other animal ownership | Presence of sheep, goats, pigs, dogs and cats at the household or neighboring farms. |
| Herd management practices | Placenta disposal methods.  Feeding placentas to dogs  Reproductive issues such as abortion history  Service type.  Introduction of new animals on to the farm. |
| Animal sampling and characteristics | Animal ID, age, dentition, breed, sex, body condition.  Calving and abortion history |
| Biological Sampling | Serum and vaginal swab sample collection.  Sample barcodes and reasons for missed samples. |
| Farm proximity | GPS coordinates (latitude, longitude). |

**Supplementary Table S2:** Univariable analysis of risk factors for Q fever seropositivity (n=1,777).

| **Variable** | **Category** | **No. of observations** | **%Q fever positive** | **Odds ratio(95%CI)** | ***P-value*** |
| --- | --- | --- | --- | --- | --- |
| Introduction of new animals | No | 942 | 8.4 (79) | Ref |  |
|  | Yes | 835 | 9.0(75) | 1.07 (0.76-1.53) | 0.691 |
| Placenta disposal | Bury | 364 | 10.0 (36) | Ref |  |
|  | Not bury | 1413 | 8.4(118) | 0.84 (0.55-1.27) | 0.396 |
| Animal management | Mixture of grazing pasture and cut and carry | 801 | 8.6 (69) | Ref |  |
|  | Pasture only | 759 | 8.7 (66) | 1.03 (0.70-1.50) | 0.884 |
|  | Zero-graze only | 217 | 8.8 (19) | 1.04 (0.59-1.83) | 0.897 |
| Animal age | 12-36 months | 450 | 8.4 (38) | Ref |  |
|  | 37-53 months | 448 | 8.0 (36) | 0.93 (0.57-1.52) | 0.785 |
|  | 54-72 months | 488 | 10.7 (52) | 1.28 (0.82-2.01) | 0.281 |
|  | 73-180 months | 391 | 7.2 (28) | 0.81 (0.48-1.37) | 0.435 |
| Animal breed | Ayrshire cross | 631 | 8.9 (56) | Ref |  |
|  | Channel Island cross | 83 | 12.0 (10) | 1.38 (0.66-2.88) | 0.393 |
|  | Holstein Friesian | 1,115 | 8.3 (93) | 0.93 (0.65-1.32) | 0.673 |

**Supplementary Table S3:** qPCR positive samples with their respective CT values.

|  | **Sample Barcode** | **Q fever CT value** |
| --- | --- | --- |
| 1. | WFN000318 | 38.74928 |
| 2. | WFN000408 | 37.33769 |
| 3. | WFN000478 | 37.05000 |
| 4. | WFN000504 | 37.56536 |
| 5. | WFN000518 | 39.93700 |
| 6. | WFN000550 | 39.63861 |
| 7. | WFN000954 | 33.83104 |
| 8. | WFN000955 | 36.34641 |
| 9. | WFN000953 | 29.18326 |
| 10. | WFN000973 | 39.96820 |
| 11. | WFN000969 | 39.30563 |
| 12. | WFN001068 | 33.11671 |
| 13. | WFN000452 | 36.01691 |
| 14. | WFN000672 | 38.79100 |
| 15.. | WFN001292 | 39.07100 |
| 16. | WFN001183 | 38.70348 |
| 17. | WFN001372 | 38.40990 |
| 18. | WFN001007 | 38.52988 |
| 19. | WFN001864 | 22.40507 |
| 20. | WFN001654 | 37.51236 |
| 21. | WFN001877 | 36.67407 |
| 22. | WFN001914 | 37.73060 |
| 23. | WFN000272 | 34.43241 |
| 24. | WFN001174 | 38.96777 |
| 25. | WFN001101 | 38.47241 |
| 26. | WFN001130 | 37.67546 |
| 27. | WFN001968 | 37.42413 |
| 28. | Positive control | 35.32100 |
| 29. | Negative control | Undetermined |
